# MDTS: automatic complex materials design using Monte Carlo tree search

**DOI:** 10.1080/14686996.2017.1344083

**Published:** 2017-07-20

**Authors:** Thaer M. Dieb, Shenghong Ju, Kazuki Yoshizoe, Zhufeng Hou, Junichiro Shiomi, Koji Tsuda

**Affiliations:** ^a^ National Institute for Materials Science, Tsukuba, Japan.; ^b^ Graduate School of Frontier Sciences, The University of Tokyo, Kashiwa, Japan.; ^c^ Department of Mechanical Engineering, The University of Tokyo, Tokyo, Japan.; ^d^ RIKEN, Center for Advanced Intelligence Project, Tokyo, Japan.

**Keywords:** Materials informatics, Materials design, Monte Carlo tree search, Si-Ge alloy interfacial structure, Python library

## Abstract

Complex materials design is often represented as a black-box combinatorial optimization problem. In this paper, we present a novel python library called MDTS (Materials Design using Tree Search). Our algorithm employs a Monte Carlo tree search approach, which has shown exceptional performance in computer Go game. Unlike evolutionary algorithms that require user intervention to set parameters appropriately, MDTS has no tuning parameters and works autonomously in various problems. In comparison to a Bayesian optimization package, our algorithm showed competitive search efficiency and superior scalability. We succeeded in designing large Silicon-Germanium (Si-Ge) alloy structures that Bayesian optimization could not deal with due to excessive computational cost. MDTS is available at https://github.com/tsudalab/MDTS.

## Graphical Abstract

**Figure:**
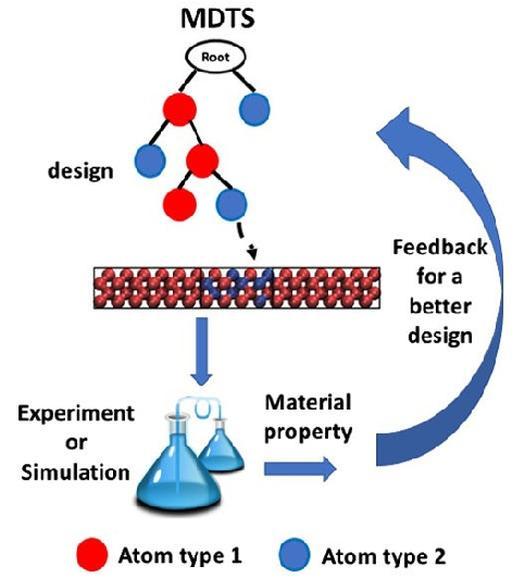

## 1. Introduction

Complex materials design is a key topic in materials science and engineering. The design of a complex materials’ structure that meets certain criteria is often formulated as the problem of finding the optimal solution from a space of candidates [1,2]. A common problem in solid-state materials design is the structure determination of a substitutional alloys problem [3,4], where atoms or vacancies are assigned to positions in a crystal structure. For example, Ju et al. [4] recently solved the optimal assignments of Silicon (Si) and Germanium (Ge) to a certain crystal structure that achieves minimum and maximum thermal conductance.

**Figure 1.**
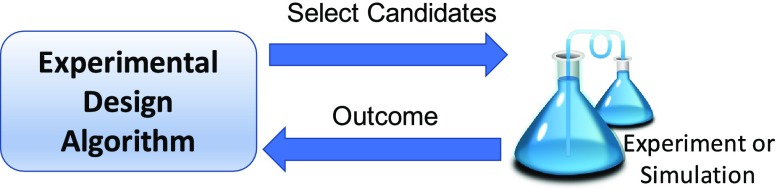
Materials design by an experimental design algorithm. The process starts with an initial random design. The algorithm selects the next candidates for experiments, where the outcome of the experiments are exploited by the algorithm to make further selection.

To accelerate the materials design process, several experimental design algorithms have been used to find the optimal structure with as few experiments as possible (Figure 1). Experimental design is an iterative process for selecting the next candidates for experiments, where the outcome of the experiments are exploited for making further choices. In many cases, simulators are substituted to experiments, e.g. first-principle calculations. In earlier studies, quantitative structure-property relationship (QSAR) models were mainly used [5]. Recently, Bayesian optimization [6], a technique to select promising candidates using Bayesian learning, has been proven as an effective tool in materials design [1,2,4,7,9]. The difference between Bayesian optimization methods and traditional QSAR models is that the uncertainty of prediction is quantified as predictive variance: the candidates are scored by an acquisition function that takes into account both predicted merit and uncertainty. Bayesian optimization is very effective in finding optimal structures but has problems with scalability, as the acquisition function has to be applied to all candidates. Evolutionary algorithms such as genetic algorithms [10,11] are more scalable, but have many parameters, such as crossover and mutation rates, that must be tuned properly to obtain the bestperformance. In most cases, in materials design, the amount of data available a priori is very limited, so tuning parameters using data may not be possible.

In this paper, we propose a novel python library called Materials Design using Tree Search (MDTS). MDTS solves structure determination of substitutional alloys with composition constraints using a Monte Carlo tree search [12], a guided-random best-first search method that showed significant success in computer Go [12,13]. Our library is highly scalable and does not have any tuning parameter.

In experiments, we applied MDTS and an efficient Bayesian optimization implementation [7] to a Si-Ge alloy interface design between two Si leads [4]. The local force field (bonding characteristics) in the structure can change due to substitution. However, in this demonstration case, we did not consider structure relaxation because the force constants of Si and Ge are known to be transferable [14]. On the other hand, there are ways to include the change in the local force constants and the current method can be simply used to incorporate such an effect [15]. The total computational time is decomposed into design time and simulation time. The former represents the selection of the next candidates and the latter simulator time. In terms of the number of calculations to find the optimal solution, Bayesian optimization was better due to its high prediction ability. However, MDTS was comparable or better in terms of total computational time, because Bayesian optimization takes exponential design time with respect to the number of atoms. MDTS is a practical tool that material scientists can easily deploy in their own problems and has the potential to become a standard choice.

## 2. Method

Consider a black-box function, 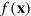, where 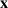 is a vector of discrete variables 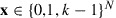. We aim to find the optimal solution 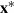 that maximizes 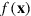 subject to composition constraints(1)
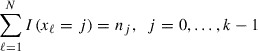



where *I* is the indicator function that returns one if the given condition is satisfied and zero otherwise. The constant 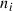 indicates the number of variables with value *i*. Notice that 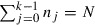. In an atom assignment problem, 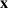 corresponds to atom types and 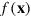 is a target property evaluated, for example, through first-principles calculations.

**Figure 2.**
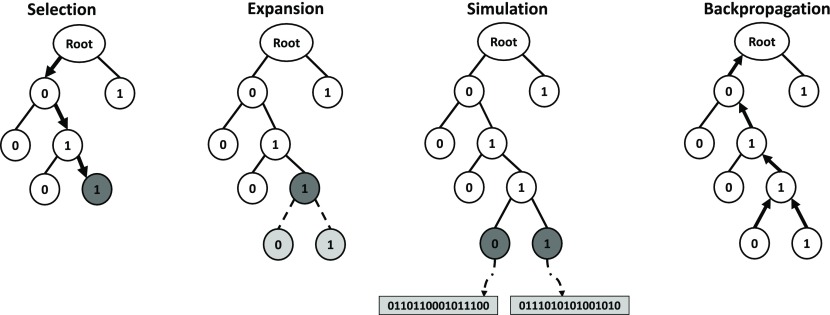
Monte Carlo tree search (MCTS) for a binary atom assignment problem. The candidate space is represented as a tree where each node represents a possible atom assignment. One round of MCTS consists of four steps, Selection, Expansion, Simulation and Backpropagation. In the selection step, a promising leaf node is chosen by following the node with the best UCB score in each branch. The expansion step adds a number of children nodes to the selected one. In simulation, solutions are created by random playouts from the expanded nodes. The backpropagation step updates nodes’ information along the path back to the root.

Monte Carlo tree search (MCTS) employs a search tree, where nodes at the 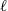
th level correspond to value assignment to 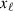 (Figure 2). A path from the root to a node at level 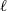 corresponds to a partial solution with respect to 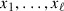. In the first round of MCTS, only the root node exists and then the search tree is gradually constructed. To obtain a full solution 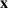, a complete path to a leaf node at the *N*th level is necessary. One interesting feature of MCTS is that only a shallow tree is built and the complete paths are obtained via random playouts [12]. A ‘playout’ creates a solution by starting from a node and determining the remaining variables randomly. The random playout allows us to explore a large candidates space without learning from data. Once a solution has been obtained by a playout, the black-box function 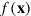 is evaluated and recorded. By combining tree expansion, backtracking and playouts, a large candidate space can be searched systematically. When a predetermined number of calculations is reached, the best solution so far is returned as the final result.

**Figure 3.**
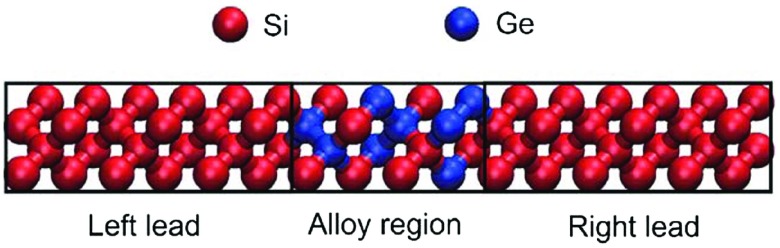
Si-Ge interfacial structure between two Si leads. In this case, the interface region is made up of 16 atoms.

Each node *i* contains three variables: the visit count 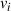 represents the number of visits in the search process; 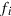 denotes the immediate merit of node *i* evaluated by playout; and the cumulative merit 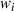 is defined as the sum of all direct merit for all descendant nodes including itself. The Upper Confidence Bound (UCB) score [12] of a node is an index indicating how promising it is to explore the subtree under the node. It is defined based on the cumulative merit and the number of visits as follows:(2)
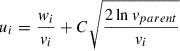



where *C* is the constant to balance exploration and exploitation and 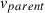 is the visit count of the parent node. Whenever a new node is added, the variables are initialized as(3)
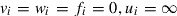



Each round of MCTS consists of: selection, expansion, simulation and back propagation (Figure 2). In the selection step, the tree is traversed from the root to a leaf by choosing the child with the maximum UCB score at each branch. If there is a tie, the winning child is chosen randomly. Let *i* denotes the identified leaf, 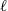 the level of the node *i*, 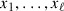 the partial solution corresponding to the path from the root to *i*. In the expansion step, children nodes are added under the node *i*. If the number of atoms *j* reaches the limit already, i.e. 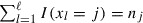 the *j*th child is not added. In the simulation step, a playout is performed from each of the added children. Notice that the random assignments are made such that the composition constraints are satisfied. With the solutions obtained, a simulator is applied to evaluate 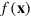 and store the value as the immediate merit of the corresponding nodes. Finally, in the back propagation step, the visit count of each ancestor node of *i* is incremented by one and the cumulative value is also updated to keep consistency.

The value of *C* crucially affects the performance of MDTS. According to the analysis by Kocsis and Szepesvári [16], to guarantee the convergence to the optimal solution, *C* should be proportional to the range between 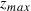 and 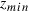, i.e. the maximum and minimum immediate merit observed in downstream nodes. Adjusting *C*, either statically or dynamically, is a standard technique for applying MCTS (as shown in [12]). Following a similar idea, MDTS controls *C* adaptively at each node as follows:(4)
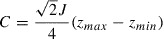



where *J* is a meta-parameter initially set to one and increased whenever the algorithm encounters a ‘dead-end’ leaf, to allow more exploration. At a dead-end leaf, the number of possible structures narrows to one. This happens when the numbers of 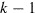 atoms reaches the limit. *J* is updated as 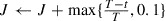, where *T* is the total number of candidates to be evaluated and *t* is the number of candidates for which the black-box function is evaluated. See supplemental material for the algorithm.

## 3. Experiments and results

In this section, we compare MDTS to a Bayesian optimization package called COMBO [7] in a binary atom assignment problem (notice that MDTS is able to handle multiple atom types assignment problems). The performance of MDTS depends on the variable ordering in 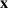. The following three options were tried: direct (left-to-right), reversed (right-to-left) and random.

**Figure 4.**
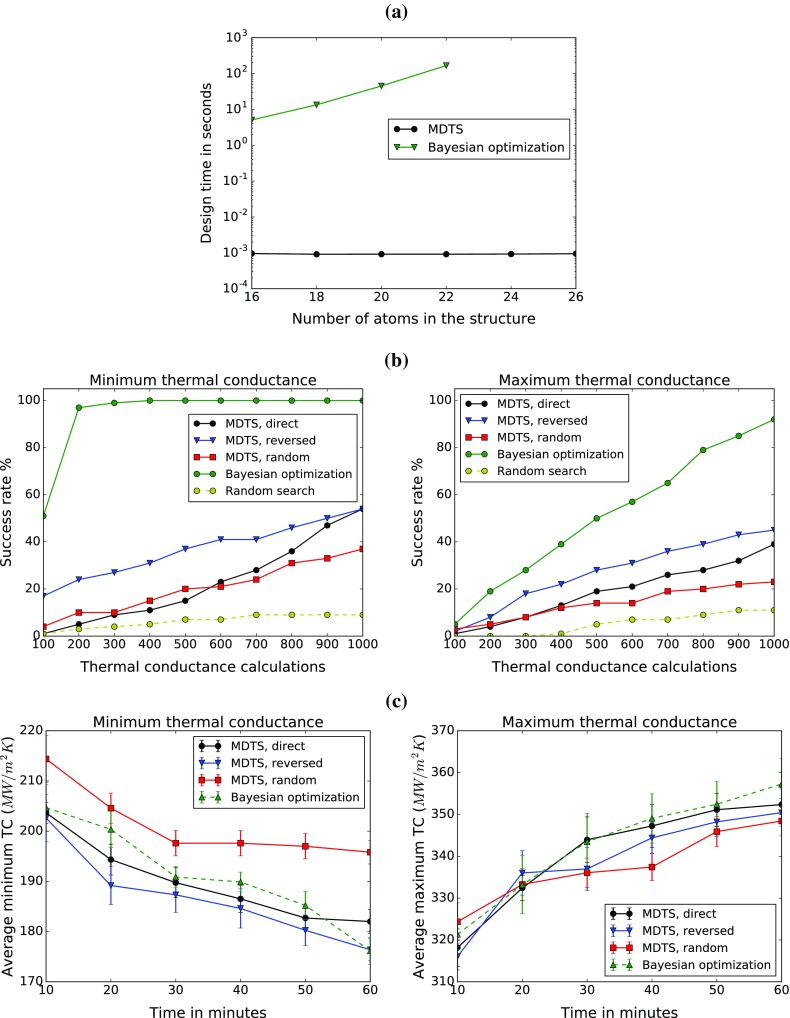
Comparison between MDTS and Bayesian optimization (BO) in finding the structure with minimum and maximum thermal conductance. (a) Design time for choosing a candidate structure against the number of atoms in the interfacial structure *N*. The time for BO grows exponentially as *N* increases. Results averaged over 10 runs, each for 30 solutions. (b) The fraction of optimal structure discovery (i.e. success rate) for both minimum and maximum thermal conductance in 100 runs against the number of thermal conductance calculations. The number of atoms is 16 (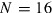). BO takes fewer calculations to find the optimal structure. (c) Optimal observed thermal conductance (minimum and maximum) against total computational time including both design and simulation time (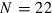). The result is averaged over 10 runs. Here, the efficiency of the two methods is comparable. For 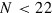, BO was more efficient and MDTS was more efficient for 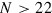.

MDTS and COMBO were applied to design optimal Si-Ge alloy (Si:Ge=1:1) interfacial structures (Figure 3) with both minimum and maximum thermal conductance [4]. Materials with both minimum (e.g. thermoelectric materials) and maximum (e.g. CPU cooling) interfacial thermal conductance have potential applications. As shown in Figure 3, the system consists of an interface region between two Si leads with infinite thickness. In the interface, there are *N* positions filled either by Si or Ge. The number of atoms of each type is constrained to *N* / 2. The number of possible structures grows rapidly as the number of atoms *N* increases. For example, at 14, 20 and 26 atoms, the number of possible structures is 3432, 184,756 and 10,400,600, respectively. The thermal conductance was computed using the atomistic Green’s function implemented in the ATK-Classical Simulator of Atomistix ToolKit (ATK) [17,18]. SiGe Tersoff [19,20] potential was used to describe the atom interactions. The size of the supercell in the transverse direction (perpendicular to the direction of heat conduction) is 1 unit cell, i.e. 5.43 Å 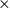 5.43 Å, and periodic boundary conditions were used. See Ref. [4] for further details.

Since the process of simulation-based structure optimization involves an experimental design algorithm and a simulation algorithm, the total computational time is divided into two parts: *design time* and *simulation time*. The design time per structure against the number of atoms is shown in Figure 4(a). Bayesian optimization shows an exponential increase in design time, because it needs to compute a score for every candidate structure. On the other hand, the design in MDTS takes only a tree traversal, whose computational cost is scarcely affected by the number of atoms. Figure 4(b) shows the fraction of optimal structure discovery over 100 runs (i.e. success rate) for both minimum and maximum thermal conductance against the number of thermal conductance calculations at 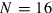. Bayesian optimization required a smaller number of calculations to achieve the same level of success rate due to its sophisticated prediction algorithm. Nevertheless, the performance of MDTS was better than random search, indicating its substantial capability of learning from data. Among the three variable orderings of MDTS, the reversed order was best. Random order performance was lowest in this particular case, likely because the existence of neighbourhood relations may be crucial for the optimal thermal conductance. Despite better learning capability, the advantage of Bayesian optimization in total computational time is rapidly wiped out, as *N* increases, because of the exponentially increasing design time. At 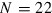, the speed of thermal conductance minimization and maximization of MDTS and Bayesian optimization is comparable as shown in Figure 4(c). At 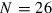, however, Bayesian optimization becomes significantly slower: it takes about 15 times more time than the 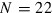 case. This result shows that MDTS should be chosen over Bayesian optimization unless the problem size is sufficiently small.

## 4. Conclusion

In this paper, we presented MDTS: a materials design library based on Monte Carlo tree search. MDTS is an open source project and interested researchers can join in the development of MDTS. The balance between design time and simulation time is an important factor in automatic materials design. Efficient design methods including MDTS are most useful when the simulation time is short. The long design time of a more inefficient machine-learning based approach can appear less problematic when the simulation time is longer. In future work, it would be necessary to pursue an adaptive approach that can balance optimality and design time in a variable manner. Additionally, we plan to make MDTS more customizable for diverse materials design problems with possibly different kinds of constraints.

## Supplementary Material

Supplementary.pdf
