# Supplementary material for: MDTS: automatic complex materials design using Monte Carlo tree search
Source: Sci Technol Adv Mater. 2017 Jul 20;18(1):498–503. doi: 10.1080/14686996.2017.1344083 (PMC5532970; doi:10.1080/14686996.2017.1344083)
Supplement: Supplementary.pdf [file tsta_a_1344083_sm1355.pdf]

Supplemental Material
MDTS: Automatic Complex Materials Design using Monte Carlo Tree Search

Thaer M. Dieb[a,b], Shenghong Ju[c], Kazuki Yoshizoe[d], Zhufeng Hou[a], Junichiro Shiomi[a,c] and Koji Tsuda[a,b,d]

[a]National Institute for Materials Science, 1-2-1 Sengen, Tsukuba, Ibaraki 305-0047 Japan; [b]Graduate School of Frontier Sciences, The University of Tokyo, 5-1-5 Kashiwanoha, Kashiwa, Chiba 277-8561, Japan; [c]Department of Mechanical Engineering, The University of Tokyo, 7-3-1 Hongo, Bunkyo, Tokyo 113-8656, Japan; [d]RIKEN, Center for Advanced Intelligence Project, 1-4-1 Nihombashi, Chuo, Tokyo 103-0027, Japan

MDTS algorithm

MDTS: Materials Design using Monte Carlo tree search is an algorithm developed to solve structure determination of substitutional alloys problem. MDTS does not need any parameter tuning and can be adapted to several problems. The candidates space is represented as a tree where each node represents a possible atom assignment. Within a predetermined number of simulations, MDTS iterates over several rounds. Each round consists of four steps, Selection, Expansion, Simulation and Backpropagation. In the selection step, a promising leaf node is chosen by following the node with the best Upper Bound Confidence (UCB) score in each branch. The expansion step adds a number of children nodes to the selected one. In simulation, solutions are created by random playouts from the expanded nodes. The backpropagation step updates nodes' information along the path back to the root.

Input: N:number of positions in the structure, AT: List of atom types, AC: List of atom constraints, T:number of calculations
Output: List of positions optimally assigned with atoms

```
function MakeNode(parent, atom)
    node ← object                          ▷ create abstract object
    node.atom ← atom                       ▷ a node is an atom assignment to a position
    node.v ← 0                             ▷ visit count
    node.f ← 0.0                           ▷ immediate merit
    node.w ← 0.0                           ▷ accumulated merit
    node.z_max ← 0.0                       ▷ maximum immediate merit observed
    node.z_min ← ∞                         ▷ minimum immediate merit observed
    node.J ← 1                             ▷ adjust hyper-parameter C
    node.parent ← parent                   ▷ node parent
    node.children ← ∅                      ▷ node children
    return node
end function
```

CONTACT Koji Tsuda. Email: tsuda@k.u-tokyo.ac.jp

```
function Select(node)
    if node has no children then
        return node
    else
        C ← (√2 node.J / 4)(node.z_max − node.z_min)
        bst_child ← argmax(node.w/node.v + C √(2ln(node.parent.v)/node.v))
        return Select(bst_child)
    end if
end function

function Expand(node)
    for all atom ∈ AT do
        child ←MakeNode(node, atom)
        node.children ← [node.children, child]
    end for
    return node.children
end function

function Simulate(node)
    structure ← list of all atoms on the path from the root to node
    if node is not maximum depth leaf then
        fill rest of the positions randomly ▷ random playout with AC ensured to be
met
    end if
    return structure
end function

function Backpropagate(node, e)
    node.w ← node.w + e
    node.v ← node.v + 1
    if e > node.z_max then
        node.z_max ← e
    end if
    if e < node.z_min then
        node.z_min ← e
    end if
    if node.parent is not None then
        return Backpropagate(node.parent, e)
    end if
end function
```

Where, in Select: $C \leftarrow \frac{\sqrt{2}node.J}{4}(node.z_{max} - node.z_{min})$ and $bst\_child \leftarrow argmax(\frac{node.w}{node.v} + C\sqrt{\frac{2ln(node.parent.v)}{node.v}})$

```
function ADJUCTC(node, j)
    node.J ← node.J + j
    if node.parent is not None then
        return ADJUCTC(node.parent, j)
    end if
end function

Start
  Tree ← MAKENODE(None, None)
  t ← 0
  allsolutions ← ∅
  while t ≤ T do
      currentnode ← SELECT(Tree)
      if currentnode is maximum depth leaf then
          solution ← SIMULATE(currentnode)
          e ← evaluate solution using experiment or computation
          BACKPROPAGATE(currentnode, e)
          allsolutions ← [allsolutions, solution]
      else
          children ← EXPAND(currentnode)
          for all child ∈ children do
              solution ← SIMULATE(child)
              e ← evaluate solution using experiment or computation
              BACKPROPAGATE(child, e)
              allsolutions ← [allsolutions, solution]
          end for
      end if
      if dead-end leaf then
          j ← max{(T−t)/T, 0.1}
          ADJUCTC(currentnode, j)
      end if
  end while
  return max(allsolutions)
End
```
